# Long noncoding RNA *TUG1* is downregulated in non-small cell lung cancer and can regulate *CELF1* on binding to PRC2

**DOI:** 10.1186/s12885-016-2569-6

**Published:** 2016-08-02

**Authors:** Pei-Chin Lin, Hsien-Da Huang, Chun-Chi Chang, Ya-Sian Chang, Ju-Chen Yen, Chien-Chih Lee, Wen-Hsin Chang, Ta-Chih Liu, Jan-Gowth Chang

**Affiliations:** 1 Graduate Institute of Clinical Medicine, Kaohsiung Medical University, No. 100, Shih-Chuan 1st Road, Kaohsiung, Taiwan; 2 Epigenome Research Center, China Medical University Hospital, No. 2, Yuh-Der Road, Taichung, Taiwan; 3 Department of Biological Science and Technology, Institute of Bioinformatics and Systems Biology, National Chiao Tung University, Hsinchu, Taiwan; 4 Department of Laboratory Medicine, China Medical University Hospital, Taichung, Taiwan; 5 School of Medicine, China Medical University, Taichung, Taiwan; 6 Division of Hematology and Oncology, Department of Internal Medicine, Kaohsiung Medical University Hospital, Kaohsiung Medical University, Kaohsiung, Taiwan; 7 Department of Laboratory Medicine, Kaohsiung Medical University Hospital, Kaohsiung Medical University, Kaohsiung, Taiwan; 8 Department of Pediatrics, School of Medicine, College of Medicine, Kaohsiung Medical University, Kaohsiung, Taiwan; 9 Division of Hematology and Oncology, Department of Pediatrics, Kaohsiung Medical University Hospital, Kaohsiung, Taiwan; 10 Division of Chest Medicine, Department of Internal Medicine, Changhua Christian Hospital, Changhua, Taiwan

**Keywords:** Long noncoding RNA (lncRNA), Taurine-upregulated gene 1 (*TUG1*), Non-small cell lung cancer (NSCLC), CUGBP and Elav-like family member 1 (*CELF1*), Circular chromosome conformation capture (4C)

## Abstract

**Background:**

Long noncoding RNAs (lncRNAs) play crucial roles in tumorigenesis, and lncRNA taurine-upregulated gene 1 (*TUG1*) has been proven to be associated with several human cancers. However, the mechanisms of *TUG1*-involved regulation remain largely unknown.

**Methods:**

We examined the expressions of *TUG1* in a cohort of 89 patients with non-small cell lung cancer (NSCLC) to determine the association between *TUG1* expression and clinical parameters. We used circular chromosome conformation capture (4C) coupled with next-generation sequencing to explore the genome regions that interact with *TUG1* and the *TUG1*-mediated regulation.

**Results:**

*TUG1* was significantly downregulated, and the *TUG1* downregulation correlated with sex (*p* = 0.006), smoking status (*p* = 0.016), and tumor differentiation grade (*p* = 0.001). Knockdown of *TUG1* significantly promoted the proliferation of NSCLC cells. According to the bioinformatic analysis result of *TUG1* 4C sequencing data, 83 candidate genes and their interaction regions were identified. Among these candidate genes, CUGBP and Elav-like family member 1 (*CELF1*) are potential targets of *TUG1* in-trans regulation. To confirm the interaction between *TUG1* and *CELF1*, relative expressions of *CELF1* were examined in *TUG1* knockdown H520 cells; results showed that *CELF1* was significantly upregulated in *TUG1* knockdown H520 cells. RNA immunoprecipitation was then performed to examine whether *TUG1* RNA was bound to PRC2, a *TUG1*-involved regulation mechanism reported in previous studies. The results demonstrated that *TUG1* RNA was bound to enhancer of zeste protein 2/embryonic ectoderm development (EZH2/EED), which is essential for PRC2. Finally, our designed ChIP assay revealed that the EZH2/EED was bound to the promotor region of *CELF1* within 992 bp upstream of the transcript start site.

**Conclusion:**

*TUG1* is downregulated in NSCLC. Using *TUG1* 4C sequencing and bioinformatic analysis, we found *CELF1* to be a potential target of *TUG1* RNA in in-trans regulation. Moreover, subsequent experiments showed that *TUG1* RNA could bind to PRC2 in the promotor region of *CELF1* and negatively regulate *CELF1* expressions in H520 cells. Our results may facilitate developing new treatment modalities targeting *TUG1*/PRC2/*CELF1* interactions in patients with NSCLC.

**Electronic supplementary material:**

The online version of this article (doi:10.1186/s12885-016-2569-6) contains supplementary material, which is available to authorized users.

## Background

As a leading cause of cancer-related mortality worldwide, lung cancer has been investigated in numerous molecular genetic studies aimed at developing new treatment strategies [1]. Lung cancer is classified into two types according to biological characteristics: non-small cell lung cancer (NSCLC) (accounting for approximately 85 % of cases), and small cell lung cancer (SCLC) [2]. The overall prognosis for lung cancer is poor; in 2004, the overall 5-year survival rate at all stages was 16.8 % [1]. Small molecule tyrosine kinase inhibitors targeting receptor tyrosine kinases (RTKs), such as epidermal growth factor receptor (EGFR) and anaplastic lymphoma kinase (ALK), play crucial roles in NSCLC treatment. Nonetheless, prognosis and outcomes for patients with certain genetic features (e.g., EGFR mutations, K-Ras mutations, and EML4-ALK rearrangement) remain poor [3].

Long noncoding RNAs (lncRNAs), defined as ncRNAs with transcripts longer than 200 nucleotides, have a critical role in the development process, cellular homeostasis, genomic imprinting, and pluripotency of embryonic stem cells [4–6]. The importance of lncRNA regulation is emphasized by their roles in the etiology human diseases [7–9]. Several lncRNAs are involved in the carcinogenesis, disease progression, or metastasis of human cancers (e.g., *MALAT1* in hepatocellular carcinoma, colorectal carcinoma, bladder cancer, and lung cancer; *HOTAIR* in breast cancer, hepatocellular carcinoma, pancreatic cancer, gastric cancer, laryngeal cancer, and nasopharyngeal cancer; *H19* in cervical, gastric, bladder, breast, esophageal, and lung cancer; *PCGEM1* in prostate cancer) [10].

The lncRNA taurine-upregulated gene 1 (*TUG1*) is a nonprotein-coding gene located on chromosome 22q12.2 that transcribes to a 6.7-kilobase-long, spliced, and polyadenylated RNA. Upregulated by taurine in developing retinal cells, *TUG1* is essential for normal photoreceptor development. Knockdown of *TUG1* leads to malformed outer segments of photoreceptors in newborn murine retinas [11]. In human cancers, *TUG1* has been reported to be associated with urothelial carcinoma of the bladder, osteosarcoma, esophageal squamous cell carcinoma, and NSCLC [12–15]. Only a few studies have proposed the mechanisms of *TUG1* regulation [15, 16]. In the present study, we examined *TUG1* expression in NSCLC patients to determine the association between *TUG1* expressions and clinical parameters. LncRNAs regulate protein-coding gene expression through chromatin remodeling, transcriptional modulation, and nuclear architecture/subnuclear localization [17]. Chromosome conformation capture (3C) techniques are methods for detecting the coassociation between chromatins through the fixation of living cells, which preserves the genomic architecture in its native state before fragmentation by restriction enzyme digestion, and the ligation of chromatin fragments that are in physical proximity in the nuclear space [18]. Circular chromosome conformation capture (4C), which involves the circularization of chimeric DNA fragments and the amplification of DNA sequences with primers within the bait but proximal to the target sequence during ligation, can be used to screen for interactions without perception of the existence of two different complexes [19]. To further investigate *TUG1* regulation, we used the 4C method to analyze genome-wide interactions with the *TUG1* gene and found a novel target of *TUG1* regulation.

## Methods

### Patient samples

Tumor and nontumor tissue samples were obtained from 89 patients with NSCLC. All participants provided written informed consent. The study was approved by the Institutional Review Board of Kaohsiung Medical University Hospital (KMUH-IRB-980524). The baseline characteristics of the patients with NSCLC (age, sex, smoking status, Eastern Cooperative Oncology Group performance status (ECOG PS), histology, differentiation grade, and TNM stage) were collected from chart records.

### RNA extraction and qRT-PCR

Total RNA extraction, complementary DNA (cDNA) generation, and polymerase chain reaction (PCR) were performed according to manufacturer protocals. The detailed procedures and primer sequences are listed in Additional file 1. Glyceraldehyde-3-phosphate dehydrogenase (GAPDH) was used as an internal control.

### Cell culture and shRNA transfection

H520, H1299, and REH cells were cultured in RPMI medium (GIBCO BRL, Gaithersburg, MD, USA) supplemented with 10 % fetal bovine serum (GIBCO BRL) at 37 °C in a 5 % CO_2_ atmosphere. *TUG1* shRNA, scramble RNA, and mock were obtained from the National Research Program for Biopharmaceuticals and were transfected into H520 and H1299 cells (400 ng *TUG1* shRNA added to 600 μL of cells, 2 × 10^5^ cells/mL) by using Lipofectamine 2000 (Life Technologies). Transfection efficiency was determined through quantitative reverse transcription polymerase chain reaction (qRT-PCR).

### Cell proliferation assay

Cell proliferation assay was used to examine whether *TUG1* knockdown affects the viability of NSCLC cells. In brief, transfected H520 and H1299 cells were plated in 96-well plates (2 × 10^5^ cells/mL, 100 μL/well). After 48 h, cell proliferation and viability were examined using the MTT assay. All experiments were performed in triplicate.

### Circular chromosome conformation capture

The 4C experiment included the following basic procedures: formaldehyde cross-linking, and digestion and ligation of the known bait chromatin (i.e., *TUG1* in this study) and the unknown sequences. Circular chimeric chromatin was then decross-linked, and the unknown sequences were amplified with inverse PCR by using the bait-specific primers. We followed the 4C method described by Stadhouders et al. [18], which involved secondary digestion and ligation between decross-linked and inverse PCR amplification. Secondary digestion is advantageous because it decreases the size of the DNA circles, enabling efficient PCR amplification of fragments. Six-base-recognizing (six-cutter) enzymes, which perform well on cross-linked chromatin, are generally recommended for primary digestion; any four-cutter enzyme that is insensitive to mammalian DNA methylation and has high religation efficiency can be used for secondary digestion. In addition, the final combination of primary and secondary restriction enzymes generates a suitable bait fragment for designing bait-specific primers for inverse PCR, depending on their compatibility [18]. Based on our bait gene sequence (*TUG1*), a six-cutter enzyme (HindIII) was used as the primary digestive enzyme, and a four-cutter enzyme (CviQI) that can generate a bait sequence suitable for further primer design in HindIII-digested *TUG1* fragments was used as the secondary digestive enzyme. In brief, REH cells were cross-linked with 1 % formaldehyde for 10 min at room temperature to preserve the three-dimensional nuclear architecture. HindIII was used to digest cross-linked chromatin (primary digestion). The digested chromatin was then ligated using the T4 DNA ligase. The digested and ligated chromatin was then decross-linked and submitted to the second restriction digestion by using CviQI to reduce the size of the fragments. Inverse PCR reactions were performed using *TUG1*-specific primers harboring Illumina adapter sequences to amplify the genomic DNA fragments ligated to *TUG1* (first PCR: forward 5’-gtctccgatagtgcacacagc-3’, reverse 5’-gaccatctccttcaggacca-3’; nested PCR: forward 5’-cattcagccaatcacaaagct-3’, reverse 5’-cagatttatgacatagttccttccaa-3’).

### Next-generation sequencing

The PCR products were purified using the Qiagen Mini-Elute kit. After purification, the amplicon was prepared for sequencing by using a Truseq DNA library preparation kit (Illumina). According to the TruSeq DNA Sample Preparation protocol, 100-ng purified amplicon pools were repaired to generate blunt-ended, 5’-phosphorylated DNA, and an A-tailing reaction compatible with the adapter ligation strategy was performed. The ligation product was purified by sample purification beads. To enrich the library, an enhanced PCR mix was used to perform PCR amplification. The size distribution of the library was verified using the High-Sensitivity DNA Kit (Agilent), and the concentration of the library was quantified using the GeneRead Library Quant Kit (Qiagen). The library was diluted and sequenced with 500 paired-end cycles on the Illumina MiSeq by following the standard protocol.

### Bioinformatic analysis of TUG1 4C-sequencing data

#### Removing known fragments from sequencing reads

Removal of known fragments from sequencing reads involved three steps. First, forward and reverse sequencing reads were merged into one sequence when the length of the overlapping region between the forward and reverse sequencing reads was more than 20 nt. Second, BLAST, a widely used bioinformatic software of sequence searching, was used to identify the location of the known fragments and primers [20]. The known fragments were located in the regions between the primers and the cutting site of the enzymes. The alignment similarity for BLAST was set at 95 %. Finally, results from Blast showed the location of known fragments and primers. These primers and known fragments of sequencing reads were removed from the sequencing reads. The remaining region of the sequencing reads was labeled “unknown fragments.”

#### Identifying potential TUG1 interaction regions

The Bowtie2 software is an efficient tool for aligning sequencing reads against reference sequences [21]. Bowtie2 was used to align unknown fragments against human genome sequences (Grch38.p2 was used in the present study). Subsequently, fourSig, a software suite for analyzing and visualizing 4C-seq data, was used to identify the potential *TUG1* interactive regions [22]. The command “bamToReTab.pl –H 210 500 300 bowtie2_output hind3_site.txt nla3_site.txt NONE > foursig_output” was used to obtain the primary results from fourSig. The primary results were then used to scan for potential interaction regions. The window size was set at 50 (i.e., 50 fragments in the window). After scanning for the interaction regions, fourSig provided three categories of regions, which were defined as potential *TUG1* interaction regions. The regions and their associated genes were listed and then annotated according to the genomic location of known human genes. These *TUG1* interactive regions, having over 100 reads of coverage, were used as *TUG1* interaction region candidates.

### RNA immunoprecipitation (RIP)

RNA immunoprecipitation (RIP) assays were performed using ChIP-IT (Active Motif, Carlsbad, CA, USA), according to the Active Motif protocol. Anti-EED (Aviva Systems Biology, San Diego, CA, USA), anti-EZH2 (Cell Signaling Technology, Danvers, MA, USA) antibodies, and IgG were used. The detailed procedures are listed in Additional file 2.

### DNA ChIP

DNA ChIP assays were performed using ChIP-IT (Active Motif, Carlsbad, CA, USA), according to manufacturer instructions. Anti-EED (Aviva Systems Biology, San Diego, CA, USA), anti-EZH2 (Cell Signaling Technology, Danvers, MA, USA) antibodies, and IgG were used. The detailed procedures are listed in Additional file 2. Six sets of primers were designed to amplify the *CELF1* promotor region. The primer sequences are listed in Additional file 3: Table S1.

### Statistical analysis

The differences in RNA expression between tumor and nontumor tissues from NSCLC patients were analyzed using a paired *t* test. The association between relative *TUG1* RNA expression levels and clinical parameters (age, sex, smoking status, ECOG PS, histology, differentiation grade, and TNM stage) was analyzed using a *t* test and ANOVA. *TUG1* shRNA and scramble shRNA transfected cells in the MTT assay were compared and analyzed through an independent samples *t* test. Statistical analyses were performed using SAS version 9.3; *p* < 0.05 was considered statistically significant.

## Results

### *TUG1* is downregulated in NSCLC tissues and affects NSCLC cell prolifiration

**Fig. 1:**
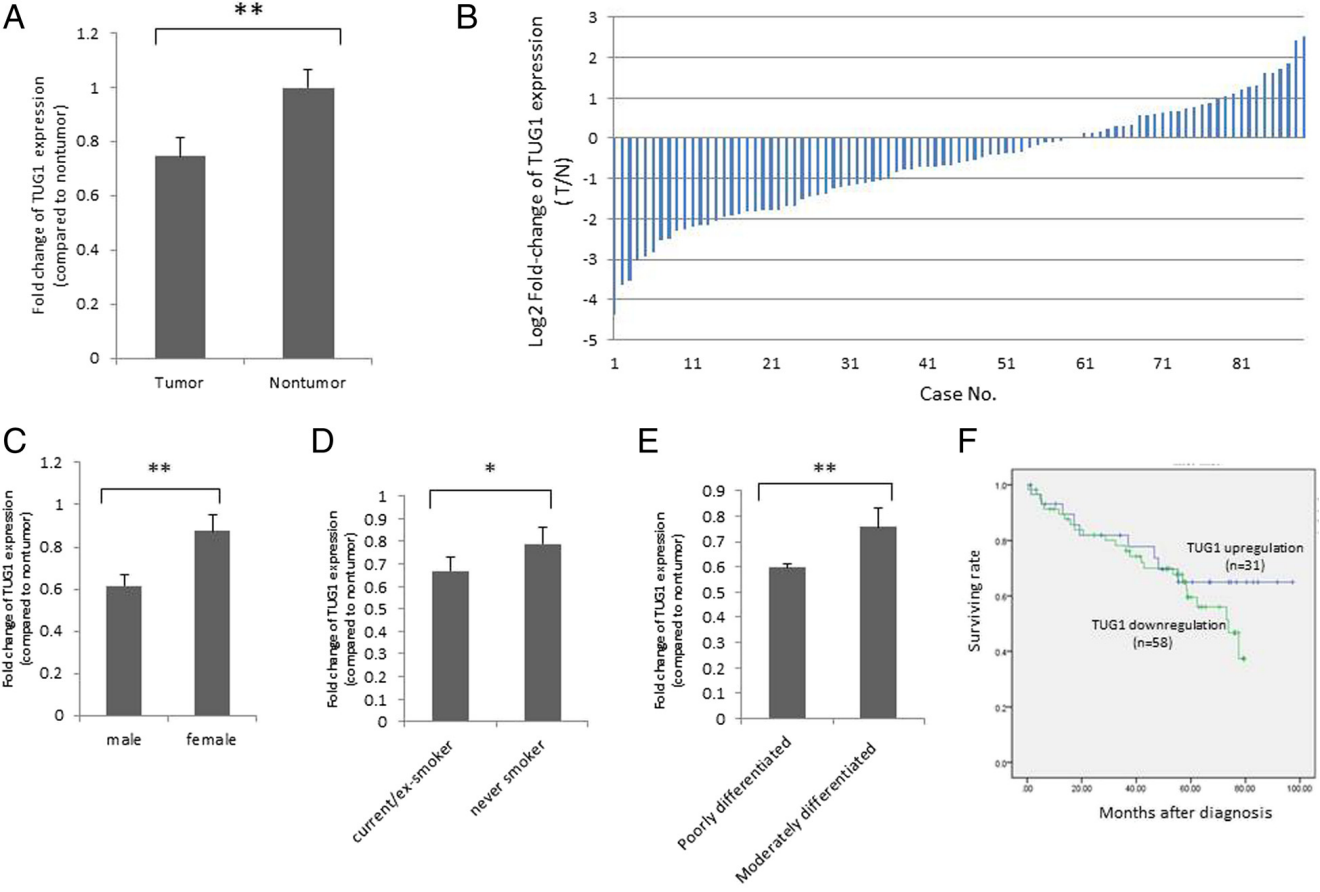
*TUG1* expressions in NSCLC lung tissues. **a** *TUG1* was examined in 89 pairs of NSCLC tumor and nontumor tissues through qRT-PCR. Fold changes in *TUG1* expression were demonstrated, revealing significant downregulation of *TUG1* in tumor tissues. **b** Log2 fold changes in *TUG1* expressions (T/N) of each case are plotted. **c**–**e** *TUG1* expression levels were significantly lower in male patients, smokers, and those with poor-differentiation tumors (*p < 0.05, **p < 0.01). **f** Survival curves for patients with *TUG1* downregulation (*n* = 58) and *TUG1* upregulation (*n* = 31)

**Fig. 2:**
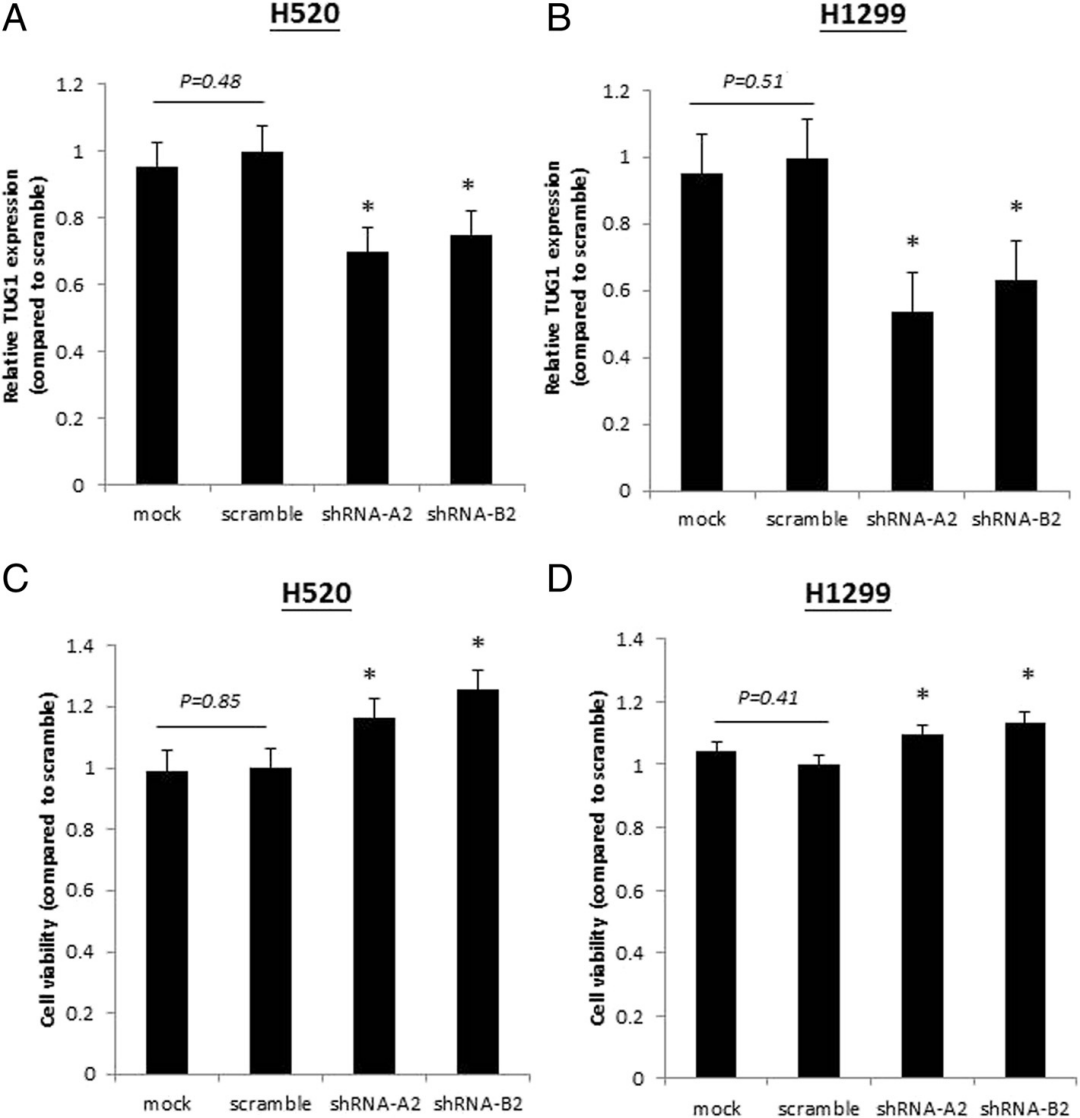
Knockdown of *TUG1* increases cell proliferation in NSCLC cells. NSCLC cells were transfected with shRNA A2 and B2 for the *TUG1* knockdown, and scramble RNA was used as the control. The efficiencies of the *TUG1* knockdown were assessed through real-time qRT-PCR in three independent experiments. **a** and (**b**) shRNA A2 and B2 demonstrated effective knockdown efficacy in H520 and H1299 cells. **c** and (**d**) Cell viabilities were examined in shRNA A2 and B2 knockdown H520 and H1299 cells. Significantly increased cell viabilities were noted compared with the scramble RNA in the three independent experiments (*p < 0.05)

We determined the levels of *TUG1* expression in 89 pairs of NSCLC tumor and nontumor lung tissues by using qRT-PCR. *TUG1* was significantly downregulated in the cancerous tissues compared with the normal counterparts (*p* < 0.01) (Fig. 1a). Over 50 % reduction in *TUG1* expression was observed in 60 % (35/58) of the *TUG1-*downregulated NSCLC tissues (Fig. 1b). Moreover, *TUG1* downregulation correlated significantly with sex (*p* = 0.006), smoking status (*p* = 0.016), and tumor differentiation grade (*p* = 0.001) (Fig. 1c–e). Correlations with the other parameters (i.e., histology, age (<65 and ≥65 years), and TNM stage) were nonsignificant (Additional file 4: Table S2). No differences were observed in the survival rates between patients with *TUG1* downregulation and those with *TUG1* upregulation until 60 months after diagnosis, indicating that survival is lower among *TUG1-*downregulated patients compared with *TUG1-*upregulated patients (Fig 1f). To explore the effects of *TUG1* regulation on cell proliferation, *TUG1* knockdown was performed using shRNAs transfection in H520 and H1299 cells. The knockdown efficiencies were validated through qRT-PCR (Fig. 2a and b). The MTT assay showed that the *TUG1* knockdown increased the cell proliferation in the H520 and H1299 cells (Fig. 2c and d).

### *CELF1* is a potential target of *TUG1* interaction and could be negatively regulated by *TUG1 RNA*

**Fig. 3:**
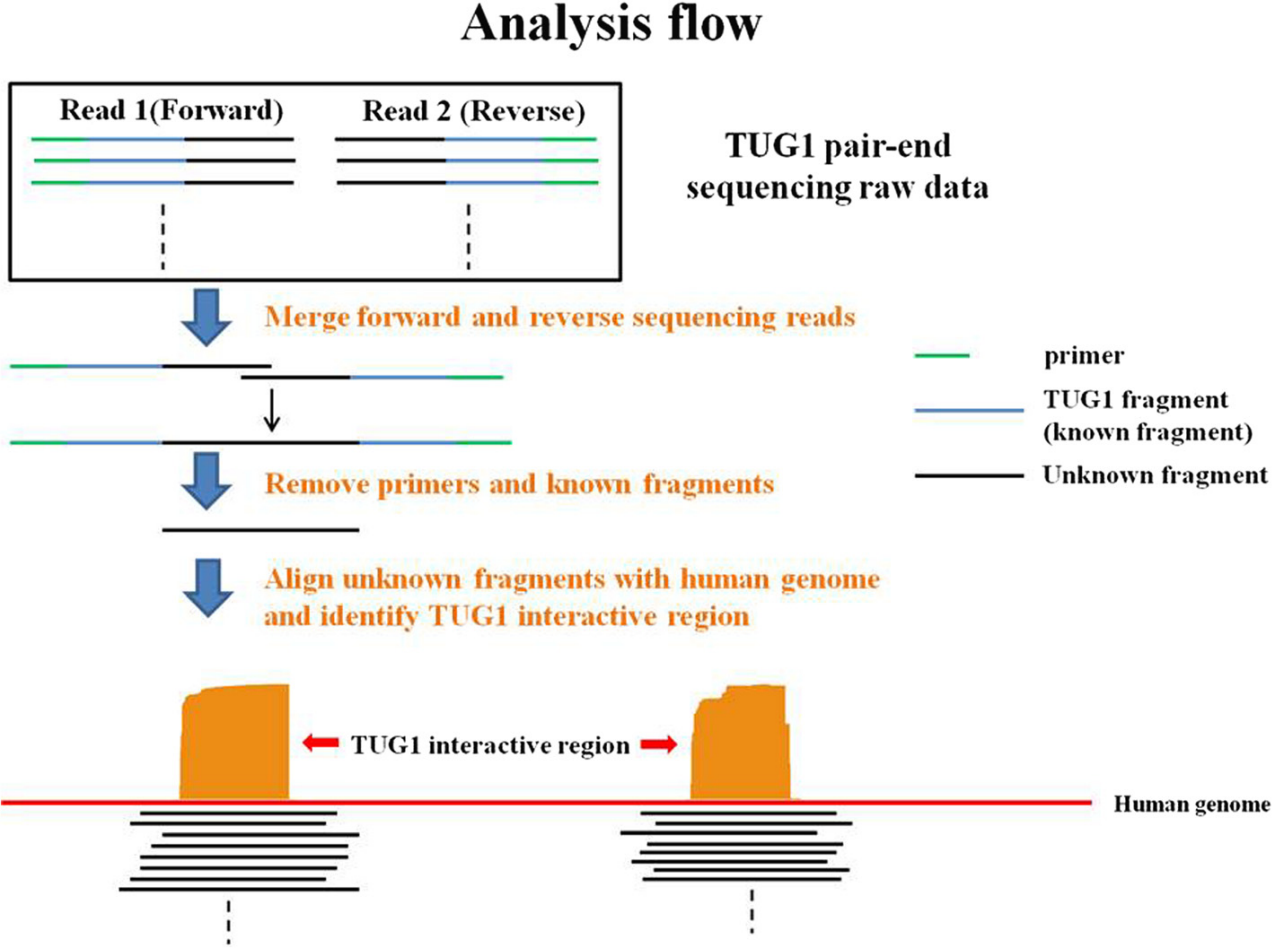
Analysis flowchart for the *TUG1* 4C-sequencing data

**Fig. 4:**
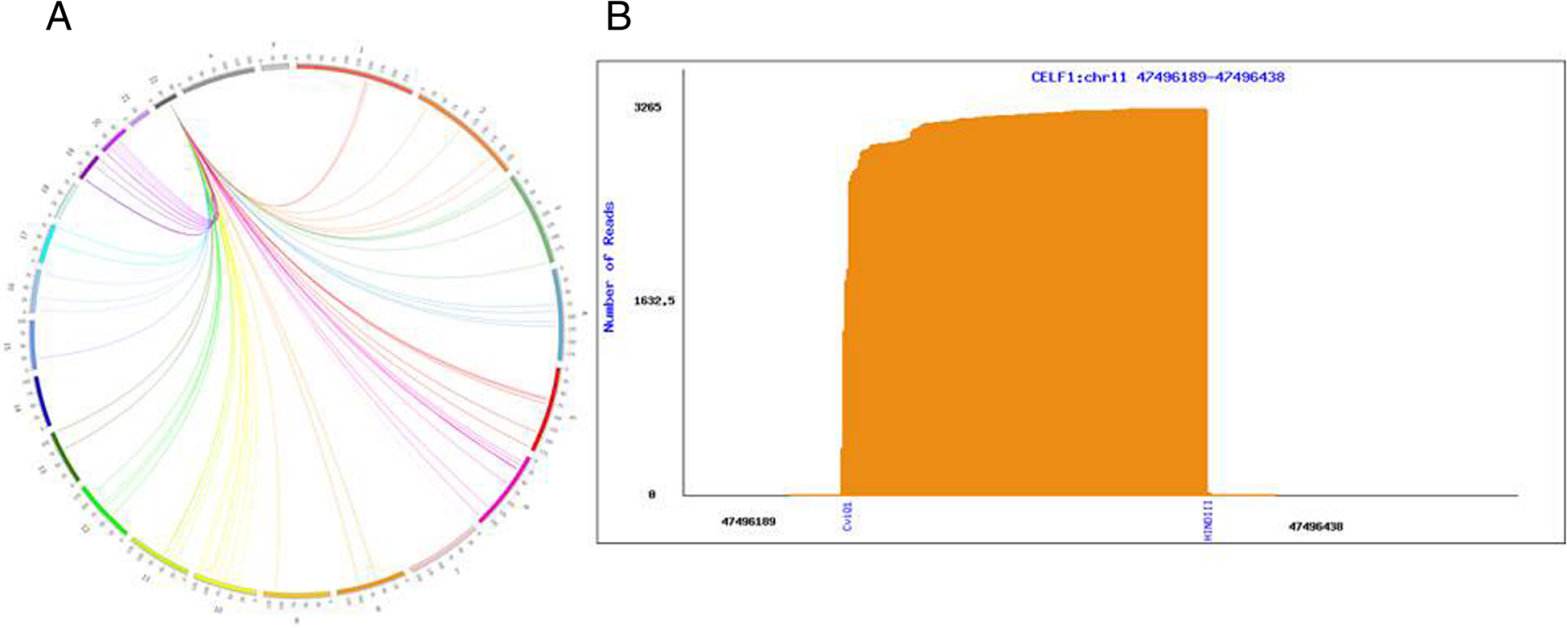
*TUG1* interaction map and read distribution of the *TUG1* interaction region (*CELF1*). **a** Circos plots depicting transinteractions and a compact representation of the interactions with other chromosomes. **b** The interaction region of *CELF1* was located at chr11:47496189-47496438. HindIII and CviQI digest sites were located at chr11: 47496402 and chr11: 47496402, respectively

**Fig. 5:**
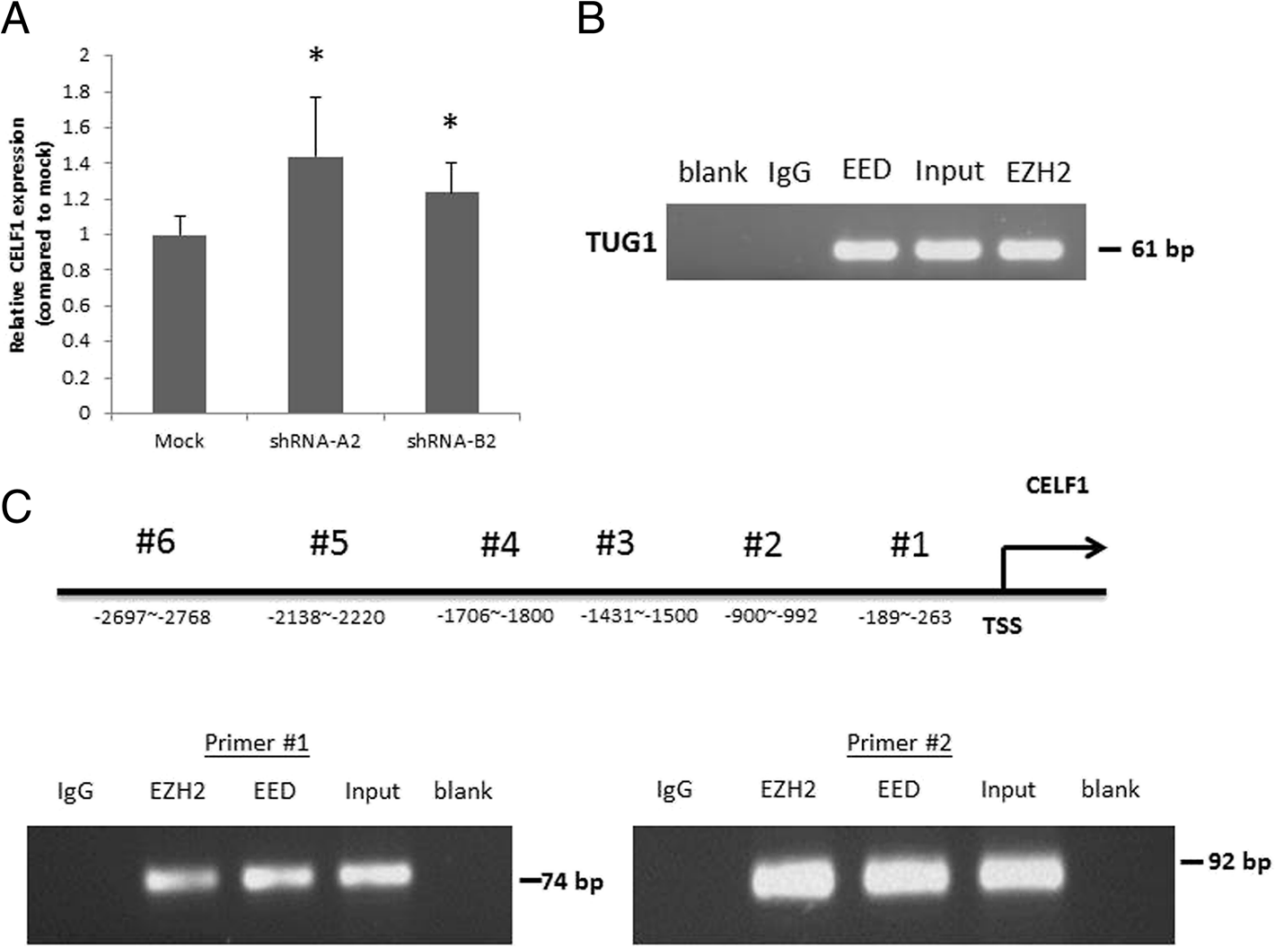
Validation of interactions between *CELF1* and *TUG1*. **a** Relative expressions of *CELF1* in the mock, shRNA-A2, or shRNA-B2 transfected H520 cells were detected through qRT-PCR in three independent experiments. Significant *CELF1* upregulation was noted in the *TUG1* knockdown H520 cells. (*p < 0.05) (**b**) RIP experiments were performed on H520 cells by using EED, EZH2, and IgG. Coprecipitated RNA was tested through qRT-PCR for *TUG1*. **c** Six primer sets were designed for ChIP experiments on EZH2 and EED of the promotor regions of *CELF1*. qPCR was used to determine the quantitation of ChIP assays. Representative images of the three independent experiments are shown

LncRNAs have been shown to regulate their target genes by physically connecting their genomic locus with the genomic regions of the target genes [23]. To further investigate *TUG1*-involved regulation, we applied 4C coupled with next-generation sequencing procedures to identify the *TUG1* interaction regions. The 4C experiment is a 3C-based technique. 3C-based techniques have been applied in investigating lncRNA regulation mechanisms. Using high-throughput chromosome conformation capture, Wang et al. reported the necessity of induced proximity between the genomic region of HOTTIP and WDR5 in HOTTIP RNA regulation of its target genes [24]. Agarose gel electrophoresis of formaldehyde cross-linked, restriction-enzyme-digested chromatin DNA, and PCR products amplified using *TUG1*-specific primers are illustrated in Additional file 5: Figure S1. The *TUG1* 4C-sequencing data had 6,302,180 paired-end sequencing reads. The data were deposited in NCBI SRA, project number: PRJNA293783. Figure 3 shows the analysis flowchart of the *TUG1* data. According to the analysis results, 83 candidate genes were identified. These genes and their interaction regions are listed in Additional file 6: Tables S3 and S4. The *TUG1* interaction map within these candidate genes is shown in Fig. 4a. Every candidate interaction region was examined to determine whether Hind III and CviQI digest sites were close to the 5’ and 3’ end of the region (Additional file 6: Table S5). Among the remaining candidate genes, the protein-coding gene *CELF1* had one of the highest abundances. Because the other protein coding gene, *MORC2*, was located in the same chromosome as *TUG1*, the possibility of self-ligation cannot be excluded. Moreover, previous studies have reported that *TUG1* regulation is involved in in-trans regulation. Therefore, an additional experiment was performed on *CELF1* to confirm the interaction between *TUG1* and *CELF1*. The interaction region of *CELF1* located at chr11:47496189–47496438, and the read distribution of *CELF1* are illustrated in Fig. 4b. The levels of *CELF1* expression were detected using qRT-PCR in the *TUG1* knockdown H520 cells. *CELF1* was found to be significantly upregulated in these cells (*p* < 0.05, Fig. 5a). Because lncRNAs might be involved in the gene regulatory network by guiding chromatin-modifying complexes to their sites of action, some lncRNAs may be central to the epigenetic control of gene expression [25]. *TUG1* was previously shown to bind to PRC2 and epigenetically repress the expression of genes involved in cell-cycle regulation [26]. Yang et al. reported that *TUG1* represses E2F-regulated growth-control genes (*MCM3*, *PCNA*, and *MSH2*) by relocating them to polycomb bodies [16]. Zhang et al. screened the HOX family in *TUG1* knockdown NSCLC cells, finding that *TUG1* can negatively regulate *HOX7* by binding to PRC2 [15]. Therefore, we investigated whether PRC2 is involved in the regulation of *TUG1* on *CELF1*. We used the RIP assay to examine the association of PRC2 and *TUG1* RNA. The binding of *TUG1* RNA and enhancer of zeste protein 2/embryonic ectoderm development (EZH2/EED), which is crucial for PRC2, was validated in the RIP assay (Fig. 5b). Furthermore, we designed six sets of primers for the promotor region of *CELF1* and found that EZH2/EED was bound to the sequences within 992 bp upstream of the transcript start site (Fig. 5c). The spatial proximity of *TUG1* and *CELF1* chromatin segments was revealed through *TUG1* 4C-sequencing and bioinformatic analysis. The interaction between *TUG1* RNA and *CELF1* was confirmed by the negative regulation of *TUG1* RNA on *CELF1* and the occupancy of *TUG1* RNA/PRC2 in the promoter region of *CELF1*.

## Discussion

*TUG1* was first identified as a noncoding RNA upregulated by taurine in developing retinal cells. Several genes related to cell-death pathways (*AIF*, *Alix/AIP1*, *NIP3*, and *NAPOR*) were shown to be upregulated in *TUG1* knockdown cells and apoptosis was found to increase, suggesting that *TUG1* is involved in cell survival [11]. Studies on human cancers have concluded that *TUG1* is overexpressed in urothelial carcinoma of the bladder and is associated with high-grade and advanced-stage diseases. Inhibition of cell proliferation and induction of apoptosis were observed in *TUG1* knockdown bladder urothelial carcinoma T24 and 5637 cells [12]. Similar results have been noted in osteosarcoma and esophageal squamous cell carcinomas [13, 14]. In lung cancer, *TUG1* was shown to be downregulated in NSCLC, and *TUG1* expression was shown to be significantly lower in advanced-stage diseases and larger tumors [15]. In our NSCLC cohort, *TUG1* was downregulated in lung cancer tissues compared with the nontumor tissues. In addition, *TUG1* levels were significanly lower in the patients who were male, smokers, or had poorly differentiated tumors. No significant difference in *TUG1* levels was observed among patients with different disease stages; this could be attributed to the limited numbers of patients with advanced-stage disease in this study (four patients with stage IV disease).

LncRNAs have been described in the processes of gene silencing, imprinting, and gene activation [8]. In our study, *CELF1* was identified through a 4C-sequencing approach; the *TUG1*-involved regulation of *CELF1* was confirmed through MTT assay, RIP, and DNA ChIP experiments. The 4C techniques, which are based on 3C techniques, enable identifying physical interactions between chromatin segments [27, 28]. In addition to 3C procedures, 4C procedures also involve a second restrictive enzyme reaction; 4C procedures introduce a circularization step after the reversal of the cross-linking, increase the likelihood of promoting intermolecular ligation events, and generate high-resolution interaction maps [29]. Previous research revealed a strong connection between the spatial organization of chromatin and gene regulation, particularly the promotor-enhancer contacts induced by chromatin looping [30]. Kaufmann et al. conducted a large-scale investigation of the interchromosomal segment and gene contact networks, and showed that coexpression and functional similarity correlate with spatial proximity [31]. In the present study, we applied 4C procedures followed by next-generation sequencing (NGS) and bioinformatic analysis to investigate the possible interactions of *TUG1* with other elements in the genome and found this approach effective for identifying lncRNA regulation targets.

*CELF1* belongs to the CELF family (CUG-BP, Elav-like family). First recognized in human cells as a nuclear RNA-binding protein, *CELF1* is expressed broadly in human tissues, such as the heart, skeletal muscles, and brain [32]. It is expressed in both the nucleus and cytoplasm, and is involved in pre-mRNA alternative splicing, RNA editing in the nucleus, and deadenylation, RNA decay, and translation in the cytoplasm [32]. *CELF1* was shown to interact with the 5’-region of *c/ebpβmRNA*, and it is associated with polysomes that translate low molecular weight isoforms of C/EBPβ [33]. In chronic myeloid leukemia, *CELF1* is repressed, resulting in a decrease of C/EBPβ isoforms, particularly LAP2. Imatinib (STI571) treatment can reinduce C/EBPβ expression that appears to depend on *CELF1* expression and the integrity of the CUG-rich intercistronic region of *c/ebpβmRNA* [34]. In oral squamous cancer cells, *CELF1* is overexpressed, and *CELF1* depletion reduces proliferation and increases apoptosis in these cells [35]. Wu et al. reported *CELF1* overexpression in NSCLC and that siRNA-mediated silencing of *CELF1* markedly reduced the survival rate and colony formation of lung cancer cells [36]. Although the roles of *CELF1* in embryonic development and carcinogenesis were revealed, the upstream regulation of *CELF1* has been overlooked. Our results reveal that *CELF1* is negatively regulated by *TUG1* in NSCLC cells. Microarray in *TUG1* knockdown murine retinal cells showed upregulation of the other members of the same CELF subfamily, *CELF2* (*NAPOR*) [11]. *CELF1* and *CELF2* exhibit conserved, partially overlapping, developmental-stage, tissue-specific expression [37]. In murine eyes, CELF1 protein expression is higher in the lens, whereas *CELF2* protein is more highly expressed in the retinae [37]. This may explain why Young et al. found no linkage between *TUG1* and *CELF1*—because that study focused on murine retinal cells. Therefore, *CELF1* and *CELF2* may share similar regulatory control mechanisms in a tissue-specific pattern through *TUG1*/PRC2 interaction.

## Conclusion

LncRNA *TUG1* is downregulated in NSCLC. We found that this downregulation is associated with sex, smoking status, and the differentiation grade of patients with NSCLC. Our study demonstrates a novel target of *TUG1* and validates the interactions of *TUG1*/PRC2/*CELF1* in NSCLC cells. The application of 4C techniques enabled a genome-wide search for lncRNA targets, the findings of which may facilitate future studies of lncRNAs. Our results may be used in the development of new treatment modalities targeting *TUG1*/PRC2/*CELF1* interactions in patients with NSCLC.

## Abbreviations

3C, chromosome conformation capture; 4C, circular chromosome conformation capture; ALK, anaplastic lymphoma kinase; cDNA, complementary DNA; CELF1, CUGBP and Elav-like family member 1; ECOG PS, Eastern Cooperative Oncology Group performance status; EED, embryonic ectoderm development; EGFR, epidermal growth factor receptor; EZH2, zeste protein 2; GAPDH, glyceraldehyde-3-phosphate dehydrogenase; lncRNAs, long noncoding RNAs; MTT, 3-(4,5-dimethylthiazol-2-yl)-2,5-diphenyltetrazolium bromide; NGS, next-generation sequencing; NSCLC, non-small cell lung cancer; PCR, polymerase chain reaction; qRT-PCR, quantitative reverse transcription polymerase chain reaction; RTK, receptor tyrosine kinase; SCLC, small-cell lung cancer; TUG1, taurine-upregulated gene 1

## Additional files

Additional file 1: RNA extraction, cDNA generation and quantitative PCR. (DOCX 19 kb) Additional file 2: RIP and DNA ChIP. (DOCX 15 kb) Additional file 3: Table S1. Sequences of primers for ChIP assay of the promoter region in *CELF1. (DOCX 19 kb)*
Additional file 4: Table S2. Clinical parameters and relative expressions of *TUG1* in NSCLC. (DOCX 17 kb) Additional file 5: Figure S1. Digestion and PCR amplification of the *TUG1*-4C procedure. (A) Agarose gel (0.8 %, wt/vol) of the undigested (left lane) and primary digested (right lane) sample (HindIII, six-cutter). (B) Agarose gel (2.0 %, wt/vol) of the secondary digested sample (CViQI, four-cutter). (C) Inverse PCR amplification of the 4C samples generated amplified sequences of a wide range of sizes. (JPG 26 kb) Additional file 6:
**Table S3.** Candidate genes of *TUG1* 4C-sequencing. **Table S4.** Gene fragments of *TUG1* 4C-sequencing. **Table S5.** Hind III and CviQI digest sites were proximal to the 5’ and 3’ end of the region. (XLSX 28 kb)
