# Supplementary material for: Long noncoding RNA TUG1 is downregulated in non-small cell lung cancer and can regulate CELF1 on binding to PRC2
Source: BMC Cancer. 2016 Aug 2;16:583. doi: 10.1186/s12885-016-2569-6 (PMC4971684; doi:10.1186/s12885-016-2569-6)
Supplement: Additional file 1: — RNA extraction, cDNA generation and quantitative PCR. (DOCX 19 kb) [file 12885_2016_2569_MOESM1_ESM.docx]

**Additional file 1.** RNA extraction, cDNA generation and quantitative PCR

Total RNAs were extracted using REzol reagent (Protech Technology Enterprise Co., Ltd.) according to manufacturer protocol, and treated with RQ1 RNase-Free DNase (Promega BioSciences, LLC. San Luis Obispo, CA, USA). Complementary DNA (cDNA) was generated from total RNAs by using the High-Capacity cDNA Reverse Transcription Kits (Applied Biosystems, Foster City, CA, USA). Quantutative polymerase chain reaction (quantitative PCR) was performed in a 10-μL reaction volume consisting of 1 μL of cDNA, 5 μL of LightCycler FastStart DNA master HybProbe (Roche Diagnostics), 0.6 μL of mixed primers, 1.2 μL of probe, and 2.2 μL of double-distilled water. The reactions were performed in the LightCycler 480 instrument (Roche Diagnostics). The PCR mixtures were preincubated for 10 minutes at 95 °C, followed by 50 cycles of 10 seconds at 95 °C, 30 seconds at 60 °C, 1 second at 72 °C, and cooled at 40 °C. The following primer pairs were used for *TUG1*, *CELF1* and *GAPDH*: *TUG1*: forward, 5’- ccagaccctcagtgcaaact-3’ and reverse, 5’- caatcaggaggcacaggac-3’; *CELF1*: forward, 5’- cagggatgcatcaccctatac-3’ and reverse, 5’- taaacagcttcctgtcttccact-3’; *GAPDH*: forward, 5’- agccacatcgctcagacac-3’ and reverse, 5’- gcccaatacgaccaaatcc-3’.
