# Supplementary material for: Long noncoding RNA TUG1 is downregulated in non-small cell lung cancer and can regulate CELF1 on binding to PRC2
Source: BMC Cancer. 2016 Aug 2;16:583. doi: 10.1186/s12885-016-2569-6 (PMC4971684; doi:10.1186/s12885-016-2569-6)
Supplement: Additional file 2: — RIP and DNA ChIP. (DOCX 15 kb) [file 12885_2016_2569_MOESM2_ESM.docx]

**Additional file 2 .** RNA immunoprecipitation (RIP) and DNA ChIP

**RNA immunoprecipitation (RIP)**

H520 cells were grown to 90 % confluence in 150-mm dishes. After cross-linking, the cells were lysed in ice-cold complete lysis buffer on ice for 30 minutes. The nuclei were pelleted at 2400 g for 10 minutes at 4 °C, and resuspended in complete shearing buffer. Chromatin was sheared into 100-bp to 1000-bp fragments by sonication, and treated with DNase I for a more efficient RIP. Ten micrograms of total chromatin was incubated overnight at 4 °C with anti-EED (Aviva Systems Biology, San Diego, CA, USA), anti-EZH2 (Cell Signaling Technology, Danvers, MA, USA) antibodies, and immunoglobulin G (IgG). After washing, the immune complex was eluted by adding 100 μL of elution buffer. Subsequently, 2 μL of 5 M NaCl was added to reverse the formaldehyde cross-linking at 65 °C for 1.5 hours. The RNA was extracted with phenol:chloroform (5:1), treated with DNase I to eliminate genomic DNA contamination, and analyzed using qRT-PCR. The PCR was performed in a final volume of 20 μL by using a LightCycler instrument (Roche Diagnostics) according to manufacturer instructions.

**DNA ChIP**

H520 cells were grown to 80 % confluence in 150-mm dishes. Chromatin was fixed by 37 % formaldehyde in FBS-free RPMI medium for 10 minutes. The fixation reaction was stopped by adding 10 mL of glycine stop-fix solution. The plates were shaken at room temperature for 5 minutes. Then the cell scraping solution was added: 10 mL of PBS, and 30 μL of 100mM PMSF. The H520 cells were pelleted by centrifugation for 10 minutes at 2500 rpm at 4 °C . The pelleted cells were resuspended and lysed in ice-cold complete lysis buffer on ice for 30 minutes. The nuclei were pelleted at 2400 g for 10 minutes at 4 °C, and resuspended in complete shearing buffer. Chromatin from H520 cells was sheared into 200-bp to 1500-bp fragments by sonication. Ten micrograms of total chromatin was incubated overnight at 4 °C with 2 μg of anti-EED (Aviva Systems Biology), anti-EZH2 (Cell Signaling Technology) antibodies, and Rabbit IgG. After washing, the immune complex was eluted by adding 50 μL of elution buffer AM2 on an end-to-end rotator for 15 minutes, before 50 μL of reverse cross-linking buffer was added. The positive control input sample consisted of 10 μg of chromitin and 2 μL of 5 M NaCl in ChIP buffer 2; the total volume was 100 μL. The ChIP and input DNA samples were incubated at 95 °C for 15 minutes. The tubes were returned to room temperature, before 2 μL of proteinase K incubate was added at 37 °C for 1 hour. Samples were already pepared for qRT-PCR analysis by using a LightCycler instrument (Roche Diagnostics), according to manufacturer instructions.
