# Supplementary material for: Long noncoding RNA TUG1 is downregulated in non-small cell lung cancer and can regulate CELF1 on binding to PRC2
Source: BMC Cancer. 2016 Aug 2;16:583. doi: 10.1186/s12885-016-2569-6 (PMC4971684; doi:10.1186/s12885-016-2569-6)
Supplement: Additional file 3: Table S1. — Sequences of primers for ChIP assay of the promoter region in CELF1. (DOCX 19 kb) [file 12885_2016_2569_MOESM3_ESM.docx]

**Additional file 3:**

**Table S1** Sequences of primers for ChIP assay of the promoter region in *CELF1*

| CELF1 promoter  Target site | Primer | Sequence (5’-3’) | Product size  (bp) |
| --- | --- | --- | --- |
| #1 | forward | tgtacactgtcagttccctggt | 74 |
|  | reverse | tccatcagatgccaaactaaag |  |
| #2 | forward | agatcaggccattacactcca | 92 |
|  | reverse | ccactcagaacataccatcttgtt |  |
| #3 | forward | gaggaggttgcagagagctg | 69 |
|  | reverse | aggctgactttcactcttattgc |  |
| #4 | forward | gcattcttaaggatcttcagacactat | 94 |
|  | reverse | cagccaatgtgctagttctcttt |  |
| #5 | forward | gctgagattacgccactgc | 82 |
|  | reverse | cgtgcccagcctatttctt |  |
| #6 | forward | aacaggaaataaaatggtttctgc | 71 |
|  | reverse | atcgggctgtatccacctct |  |
