# Supplementary material for: Long noncoding RNA TUG1 is downregulated in non-small cell lung cancer and can regulate CELF1 on binding to PRC2
Source: BMC Cancer. 2016 Aug 2;16:583. doi: 10.1186/s12885-016-2569-6 (PMC4971684; doi:10.1186/s12885-016-2569-6)
Supplement: Additional file 4: Table S2. — Clinical parameters and relative expressions of TUG1 in NSCLC. (DOCX 17 kb) [file 12885_2016_2569_MOESM4_ESM.docx]

**Additional file 4:**

**Table S2** Clinical parameters and relative expressions of *TUG1* in NSCLC

| Characteristics | | *TUG1* | |  |
| --- | --- | --- | --- | --- |
|  |  | n | △Ct  Mean ± SD |  |
| Gender | Male | 48 | 5.37 ± 1.51 | *p*=0.006 |
|  | Female | 41 | 4.50 ± 1.41 |  |
| Age | <65 y/o | 36 | 5.24 ± 1.59 | *p*=0.16 |
|  | ≥65 y/o | 53 | 4.78 ± 1.45 |  |
| Smoking | Never-smoker | 53 | 4.66 ± 1.37 | *p*=0.01 |
|  | Current/ex-smoker | 36 | 5.44 ± 1.62 |  |
| *ECOG PS | 0 | 44 | 4.71 ± 1.58 | *p*=0.15 |
|  | 1 | 44 | 5.17 ± 1.39 |  |
| Histology | Adenocarcinoma | 50 | 4.50 ± 1.44 | *p*=0.22 |
|  | Squamous cell carcinoma | 33 | 5.62 ± 1.40 |  |
|  | others | 6 | 5.35 ± 1.60 |  |
| Grade | Moderately differentiated | 76 | 4.77 ± 1.44 | *p*=0.001 |
|  | Poorly differentiated | 13 | 6.18 ± 1.47 |  |
| Stage | I | 46 | 4.66 ± 1.58 | *p*=0.48 |
|  | II | 23 | 5.42 ± 1.63 |  |
|  | III | 16 | 5.22 ± 1.12 |  |
|  | IV | 4 | 5.03 ± 0.95 |  |

NSCLC, non-small cell lung cancer; ECOG PS, Eastern Cooperative Oncology Group Performance Status; SD: standard deviation

*Only one patient was classified as 2 in ECOG PS, therefore the patient was excluded in analysis for this item.
