# Supplementary figures and images for: Long noncoding RNA TUG1 is downregulated in non-small cell lung cancer and can regulate CELF1 on binding to PRC2
Source: BMC Cancer. 2016 Aug 2;16:583. doi: 10.1186/s12885-016-2569-6 (PMC4971684; doi:10.1186/s12885-016-2569-6)

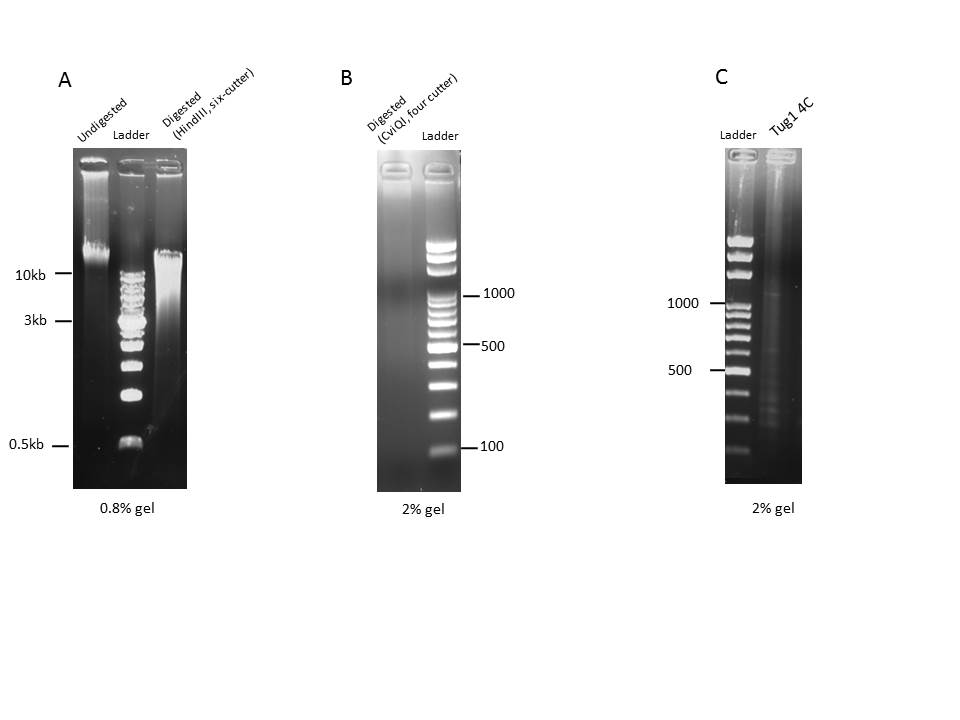

Supplement: Additional file 5: Figure S1. — Digestion and PCR amplification of the TUG1-4C procedure. (A) Agarose gel (0.8 %, wt/vol) of the undigested (left lane) and primary digested (right lane) sample (HindIII, six-cutter). (B) Agarose gel (2.0 %, wt/vol) of the secondary digested sample (CViQI, four-cutter). (C) Inverse PCR amplification of the 4C samples generated amplified sequences of a wide range of sizes. (JPG 26 kb) [file 12885_2016_2569_MOESM5_ESM.jpg]
